# Interactions Between Circulating Tumor Cells and the Immune System in Colorectal Cancer: Friends or Foes?

**DOI:** 10.3390/cancers18132104

**Published:** 2026-06-29

**Authors:** Michela De Meo, Chiara Nicolazzo

**Affiliations:** 1 Cancer Liquid Biopsy Unit, Department of Experimental Medicine, Sapienza University of Rome, 00161 Rome, Italy; 2 Department of Life Science, Health, and Health Professions, Link Campus University, 00165 Rome, Italy

**Keywords:** colorectal cancer, circulating tumor cells, immune evasion, immunotherapy, PD-L1, tumor microenvironment, metastasis, liquid biopsy, neutrophil extracellular traps, immune checkpoint

## Abstract

Colorectal cancer (CRC) is a leading cause of cancer death worldwide, mainly due to metastasis. Circulating tumor cells (CTCs) act as the biological “seeds” of dissemination, traveling through the bloodstream to colonize distant organs. However, the blood is a hostile environment where CTCs must constantly face immune pressure. This review explores the bidirectional interactions between CTCs and immune cells in CRC, asking whether CTCs are merely vulnerable targets of immunosurveillance or can exploit the immune system for survival and metastasis. We dissect intrinsic and extrinsic immune evasion mechanisms, including MHC-I modulation, immune checkpoint expression (PD-L1, CD47, FasL), platelet cloaking, and neutrophil extracellular traps (NETs). Furthermore, we examine how CTCs form heterotypic clusters with monocytes, neutrophils, and lymphocytes, creating pro-metastatic niches and promoting phenotypic plasticity. The impact of CTCs on systemic immunity, including reprogramming of NK cells, T lymphocytes, and myeloid-derived suppressor cells (MDSCs), is discussed. Importantly, we highlight the emerging role of CTCs as dynamic biomarkers for immunotherapy, focusing on the predictive value of PD-L1+ CTCs and the potential of CTC-derived neoantigens for personalized vaccination. Despite progress, challenges remain in standardization, detection sensitivity, and clinical validation. Understanding the equilibrium between immune elimination and evasion by CTCs is crucial to develop novel interventions that interrupt the metastatic dialog and improve outcomes for CRC patients.

## Simple Summary

Colorectal cancer often spreads to other organs through circulating tumor cells (CTCs) that travel in the bloodstream. Normally, the immune system should recognize and destroy these cells. However, CTCs can hide or protect themselves using several strategies. They can reduce their visible “identity tags”, display “do not kill me” signals such as PD-L1, or surround themselves with platelets and neutrophil traps to form a physical shield. CTCs can cluster with immune cells, turning them into helpers rather than attackers. This review explains how CTCs escape and manipulate the immune system in colorectal cancer. Understanding these mechanisms may help doctors predict which patients need more intensive or targeted treatments. Moreover, studying CTCs through a blood test (liquid biopsy) could guide personalized immunotherapy, such as immune checkpoint inhibitors or custom-made vaccines. Unraveling this battle between CTCs and the immune system offers new hope for stopping metastasis and improving patient survival.

## 1. Introduction

Colorectal cancer (CRC) represents one of the most frequently diagnosed neoplasms and one of the leading causes of cancer death worldwide [1]. Despite advances in screening strategies, surgery, and systemic therapies, patient prognosis remains strictly linked to the appearance of distant metastases, particularly in the liver and lungs [2,3].

Metastasis is not a random event but a highly orchestrated biological process that includes the acquisition of invasive characteristics, the degradation of the extracellular matrix, intravasation into the bloodstream or lymphatic system, survival in adverse hemodynamic conditions, and finally, extravasation and colonization of a new tissue microenvironment [3,4,5]. This pathway implies a profound phenotypic and molecular plasticity of tumor cells which must adapt to selective pressures different from those present in the primary tumor [4,5].

In this context, circulating tumor cells (CTCs) play a crucial role, configuring themselves as the biological “seed” of metastasis according to the “seed and soil” model [6,7].

CTCs represent an extremely rare and heterogeneous population, characterized by morphological, phenotypic, and functional variability [7,8,9,10]. They can present epithelial, mesenchymal, or hybrid traits, reflecting the dynamics of epithelial-to-mesenchymal transition (EMT) and its reversibility [11,12]. Beyond their prognostic and predictive relevance, CTCs constitute a real-time model of tumor evolution, as they reflect the selective pressure exerted by therapies and the systemic microenvironment [13,14]. However, the blood compartment is not simply a passive means of transport: it is a hostile environment, characterized by mechanical stress, anoikis, and, above all, constant monitoring by the immune system [8,14,15,16].

The immune system indeed plays a central role in controlling tumor progression through the process of immunosurveillance, whereby effector cells such as cytotoxic T lymphocytes (CTLs), Natural Killer (NK) cells, and macrophages recognize and eliminate transformed cells [17,18]. However, the interaction between a tumor and the immune system is not static but evolves over time according to the immunoediting paradigm, articulated in the phases of elimination, equilibrium, and escape [19,20].

In an initial phase, the most immunogenic tumor cells can be recognized and destroyed; subsequently, an equilibrium phase can establish itself, in which immune pressure selects less immunogenic clones; finally, in the escape phase, subpopulations capable of evading immune recognition emerge through mechanisms such as the downregulation of tumor antigens, expression of immunosuppressive molecules, or induction of a tolerogenic microenvironment [19,20,21].

**Figure 1:**
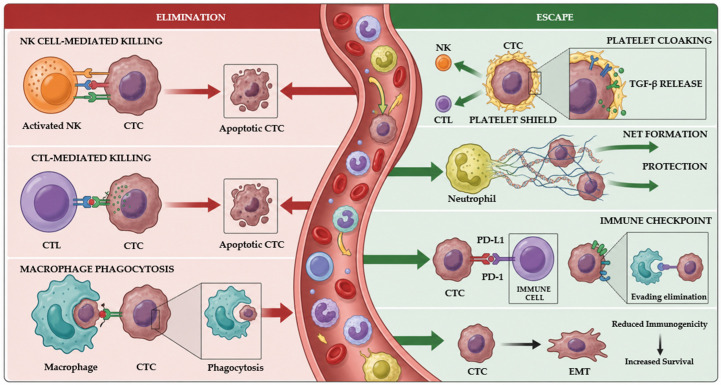
The journey of CTCs in the bloodstream: the interplay between immune elimination and evasion. The left panel illustrates the principal mechanisms involved in CTC elimination, including natural killer (NK) cell-mediated cytotoxicity, cytotoxic T lymphocyte (CTL)-mediated killing, and macrophage phagocytosis. The right panel depicts the main immune escape strategies adopted by CTC, such as platelet cloaking, neutrophil extracellular trap (NET) formation, immune checkpoint exploitation, and epithelial-to-mesenchymal transition (EMT), which collectively enhance survival, immune evasion, and metastatic dissemination.

CTCs are positioned exactly in this dynamic balance between elimination and evasion (Figure 1). During their time in circulation, they are exposed to immune cells and soluble mediators, but they can also establish protective interactions, for example by associating with platelets or modulating the expression of immune checkpoint molecules [16,22]. Therefore, a biologically and clinically relevant question arises: do CTCs simply represent vulnerable targets of systemic immune activity, or are they capable of exploiting the immune system, directly or indirectly, to increase their survival and metastatic potential? Understanding this dual nature of CTCs could provide new interpretative keys to metastatic progression in CRC and open innovative perspectives for targeted therapeutic strategies.

## 2. Immune Escape Mechanisms of CTCs in Colorectal Cancer

**Figure 2:**
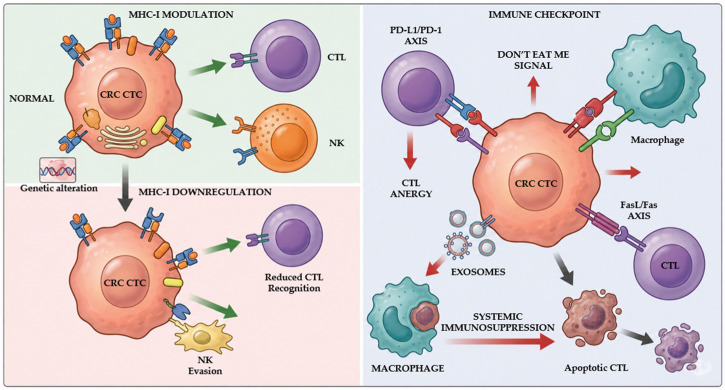
MHC-I modulation and immune checkpoint expression on CRC CTCs. The left panel illustrates the impact of major histocompatibility complex class I (MHC-I) modulation on immune cell recognition. Under physiological conditions, preserved MHC-I antigen presentation machinery, including components such as TAP1 and LMP7, supports cytotoxic T lymphocyte (CTL) recognition and activation, while also influencing natural killer (NK) cell responses. Conversely, downregulation or alteration of MHC-I and associated antigen-processing machinery reduces CTL-mediated recognition and contributes to immune escape, with concurrent modulation of NK cell susceptibility. The right panel depicts key immune checkpoint and immune evasion pathways exploited by CRC CTCs, including the PD-L1/PD-1 axis, CD47/SIRPα signaling, and FasL/Fas interactions, which collectively promote T-cell exhaustion or apoptosis, inhibit macrophage-mediated phagocytosis, and foster systemic immunosuppression, thereby enhancing CTC survival and metastatic potential.

CTCs represent an extremely heterogeneous and dynamic population, capable of surviving in the bloodstream despite constant surveillance by the immune system [16]. In CRC, several immune evasion mechanisms allow CTCs to escape recognition and destruction by immune cells, thus favoring metastatic dissemination [23] (Figure 2). Among these, modulation of antigen presentation, expression of immune checkpoint molecules, and interaction with blood components such as platelets and neutrophils are particularly relevant [22,24,25,26] (Table 1).

### 2.1. Modulation of MHC Expression and Antigen Presentation Machinery

One of the main mechanisms through which CTCs evade the immune system is the reduction in expression of major histocompatibility complex class I (MHC-I) molecules, which are essential for presenting tumor antigens to CTLs [27]. This phenomenon is often sustained by alterations in key components of the antigen processing and presentation machinery (APM), including TAP1/2, β2-microglobulin, and immunoproteasome subunits, whose loss or epigenetic repression reduces the peptide load presented on MHC-I and favors immune escape [28]. Ling et al. explored the possible molecular mechanisms involved in tumor immune evasion in CRC and found that TAP1 downregulation correlates with immune evasion and unfavorable prognosis in CRC patients. Furthermore, to understand the possible regulatory mechanisms of TAP1 expression, the relationship with DNA methylation was studied, and it emerged that most of the CpG sites associated with TAP1 that were most involved clustered in 3 distinct regions along the gene: near the promoter region upstream of exon 1, in a region encompassing exons 2 and 3, and in a region encompassing exon 11. Focusing the analysis on a total of 9 CpG sites closest to the TAP1 promoter, a statistically significant difference was highlighted regarding the methylation status among these groups, but all CpG sites showed a higher level of methylation in the group of tumors with low TAP1 expression [29].

Furthermore, microenvironmental factors such as hypoxia can coordinately reduce MHC-I, TAP1/2, and LMP7, further limiting antigen presentation [30]. In the study by Sethumadhavan et al., it was demonstrated that oxygen tension in the TME modulates MHC-I-mediated antigen presentation. In particular, hypoxia induces a downregulation of MHC-I expression in an HIF-dependent manner, as evidenced both in in vivo models and in hypoxic three-dimensional tumor cultures, but not in two-dimensional systems. Concurrently, the same study highlighted that hypoxia reduces the expression of key components of the antigen presentation machinery, including TAP1/2 and LMP7. Conversely, hyperoxia conditions promote the transcriptional upregulation of MHC and associated proteins (TAP2, LMP2/7) [30]. In the context of CRC and other solid tumors, intra-tumoral heterogeneity of MHC-I expression reflects a process of genetic and epigenetic immune editing, where clones with reduced APM/MHC-I function are selected under T-cell pressure and can dominate advanced and metastatic lesions [28,31]. The partial or reversible loss of MHC-I allows tumor cells to escape CTL surveillance while limiting NK cell activation, which recognize the complete absence of MHC-I as a “missing self” signal via inhibitory receptors such as KIR and NKG2A; for this reason, a complete loss of MHC-I is rare in solid tumors [74]. This is consistent with the hypothesis that CTCs present reduced or mosaic expression of MHC-I compared to the primary tumor, suggesting the selection of subclones more resistant to CTL surveillance during dissemination [32].

However, a complete loss of MHC-I renders cells highly susceptible to NK cell activity, which recognize the “missing self” phenotype through the absence of inhibitory MHC-I ligands for KIR/NKG2A receptors as demonstrated by Bern et al. [75]. For this reason, complete loss of MHC-I is relatively rare in solid tumors, while partial or reversible configurations are more frequent, often subclonal, allowing for escape from CTLs while limiting full NK activation [33,34]. It follows that CTCs tend to maintain sufficient MHC-I levels to provide inhibitory signals to NKs and avoid immediate cytotoxicity, but not enough to allow for effective antigen presentation and a robust CTL response, thus achieving a sophisticated form of adaptive immune escape [34].

In summary, the modulation of MHC-I expression and APM components represents a finely tuned immune evasion strategy in CRC, where CTCs exploit genetic, epigenetic, and microenvironmental mechanisms to achieve a “Goldilocks” level of MHC-I expression: sufficiently low to evade CTL-mediated killing, yet sufficiently high to avoid triggering NK cell “missing self” recognition, thereby enabling their survival during hematogenous dissemination and metastatic colonization.

### 2.2. Expression of Immune Checkpoints on CTCs

CTCs can actively contribute to immune suppression through the expression of immune checkpoint molecules, particularly Programmed Death-Ligand 1 (PD-L1) [35]. The interaction between PD-L1 expressed on CTCs and the PD-1 receptor on T lymphocytes leads to inhibition of cytotoxic activity, promoting a state of T-cell exhaustion [36]. In CRC, several pieces of evidence indicate that a subpopulation of CTCs express PD-L1, often associated with more aggressive phenotypes and EMT characteristics [36].

The foundational 2014 study by Steinert et al. demonstrated for the first time, through gene expression analysis on manually isolated CTCs from CRC patients, a marked upregulation of CD47 and transcriptional alterations compatible with a dormant state, suggesting that CTCs adopt active immune escape mechanisms to survive in the circulation [44]. A retrospective study on 182 CRC patients showed that 42.2% of CTC-positive patients expressed PD-L1 on CTCs, with association across all disease stages, including stable disease, suggesting a role as a dynamic biomarker of minimal residual disease (MRD) [37]. A broader analysis of 666 CRC patients confirmed that 74.6% of patients with CTC presented PD-L1 expression, with CTC clusters also found in 13% of cases [38]. Satelli et al. in 2016 isolated CTCs with an EMT phenotype (surface vimentin positive) from metastatic CRC patients and demonstrated that nuclear expression of PD-L1 in CTCs was significantly associated with reduced survival [39]. The association between PD-L1 and EMT in CRC was confirmed by Secinti et al.: PD-L1 expression in colorectal cancer cells correlated significantly with EMT status (*p* < 0.001), lymph node metastases, advanced stage, and reduced disease-free survival [40].

In vitro studies have demonstrated that FGFR2 induces PD-L1 expression in CRC cell lines (SW480 and NCI-H716) through the JAK/STAT3 pathway, with in vivo confirmation in murine xenograft models [41]. Furthermore, Wu et al. showed that CRC cell-derived exosomes containing miR-372-5p upregulate PD-L1 in macrophages through the PTEN/AKT/NF-κB pathway, suppressing the activity of CD3+CD8+ T lymphocytes in co-culture [42]. PD-L1 expression can be induced by inflammatory signals, such as IFN-γ, present in the tumor microenvironment and the bloodstream [43].

Beyond PD-L1, other immunoregulatory molecules such as PD-L2, CD47, and FasL can be expressed by CTCs, further contributing to their ability to evade the immune response. Regarding CD47, in 2012, Willingham demonstrated that CD47 is overexpressed in CRC tumor cells and that blocking CD47 with monoclonal antibodies restored macrophage phagocytosis in vitro and inhibited tumor growth in murine xenograft models with patient-derived tumor cells [45]. In 2023, Tang et al. discovered an additional SIRPα-independent mechanism: CD47 cis-masks the pro-phagocytic ligand SLAMF7 on the tumor surface and blocking CD47 (but not SIRPα) restores phagocytosis [46]. In 2025, Miller et al. clarified the signaling cascade downstream of SIRPα, demonstrating how CD47 inhibits Vav phosphorylation in macrophages, blocking Rac activation and IgG-mediated phagocytosis [47]. Specifically in CRC, Arai et al., analyzing 14,287 CRC cases, demonstrated that high CD47 expression is associated with CMS1 and CMS4 subtypes and the activation of oncogenic pathways (MAPK, PI3K, TGF-β) [48]. Furthermore, Kang et al. demonstrated in vivo that dual blockade of CD47 and TNFR2 in murine CRC models produces synergistic anti-tumor effects, with a reduction in Tregs and increased CD8+ activation [49].

Concerning FasL, as early as 1996, the “Fas counterattack” was demonstrated in CRC: the SW620 cell line expresses functional FasL and kills Jurkat T lymphocytes in a Fas-mediated manner in vitro, while the same tumor cells are resistant to Fas-mediated apoptosis [50]. Later, these results were extended by demonstrating that CRC cells release soluble FasL that induces dose-dependent apoptosis in Jurkat lymphocytes without cell–cell contact, and that serum levels of soluble FasL are significantly elevated in CRC patients [51]. Specifically regarding CTCs, Papadaki et al. demonstrated Fas/FasL expression on CTCs in metastatic breast cancer patients: FasL was expressed in 92.3% of CTCs, and Fas/FasL co-expression was associated with significantly reduced PFS (9.5 vs. 13.4 months; *p* = 0.009) [52].

In addition to these well-studied checkpoints, the role of soluble ligands in modulating NK cell function is critical [76]. Regarding NK cell inhibition, it would have been valuable to mention soluble ligands such as MICA and MICB [76]. The soluble forms of MICA and MICB (sMICA/sMICB) bind to the activating receptor NKG2D on NK cells, inducing its internalization and degradation [77]. The shedding of these ligands from tumor cells can lead to reduced expression of the activating receptor NKG2D on NK cells, thereby impairing their cytotoxic activity against CTCs and facilitating immune evasion [78]. In CRC, Doubrovina et al. demonstrated that NK cells from patients with elevated serum levels of sMIC exhibited reduced expression of NKG2D and CXCR1, highlighting the role of sMIC in impairing NK functions [77].

Regarding PD-L2, it functions as an immune checkpoint independent of PD-L1 and is expressed in about 40–80% of CRC patients [53]. In 2023, Lv et al. verified PD-L2 expression in tumor-associated macrophages (TAMs) in CRC using single-cell RNA sequencing (scRNA-seq), multiplex immunofluorescence, and flow cytometry. PD-L2+ TAMs showed a pro-tumoral M2 phenotype and increased the migratory, invasive, and proliferative capacity of colon cancer cells in transwell and colony formation assays [54]. In 2024, Zhu et al. conducted in vivo experiments on C57BL/6 mice with the MC38 cell line, demonstrating that targeting PD-L2 emerges as a complementary therapeutic strategy to PD-1/PD-L1 blockade. Although PD-L2 is inducible by IFN-γ like PD-L1, it shows a unique spatial distribution in the tumor microenvironment. Blockade of both checkpoints revealed a significant correlation with the infiltration of various immune cells, including multiple dendritic cell subtypes, implying enhanced antigen presentation [53]. Also, in 2024, Liu et al. demonstrated that PD-L2 is expressed mainly on tumor-derived exosomes (TDE-PD-L2) with surface localization. Under immunocompetent conditions, TDE-PD-L2 is sequestered by immune cells in a PD-1-dependent manner, systemically suppressing T cell function by increasing the proportion of Tregs and decreasing cytotoxic CD8+ T cells both in tumor-infiltrating lymphocytes and in the spleen [55].

In summary, CTCs deploy a multi-layered immune checkpoint strategy that extends well beyond PD-L1: the coordinated expression of PD-L1, PD-L2, CD47, and FasL, together with the shedding of soluble NKG2D ligands (sMICA/sMICB), enables CTCs to simultaneously suppress CTL cytotoxicity through T-cell exhaustion, evade macrophage phagocytosis via the CD47-SIRPα “don’t eat me” axis, induce apoptosis of effector lymphocytes through the Fas counterattack, and impair NK cell-mediated killing by downregulating NKG2D, thereby constructing a comprehensive immune shield that facilitates CTC survival during hematogenous dissemination and correlates with adverse clinical outcomes in CRC patients.

In conclusion, the expression of these checkpoints on CTCs not only favors survival in the circulation but also represents a potential dynamic biomarker for monitoring response to immunotherapies in CRC.

### 2.3. Extrinsic Protection Mechanisms: “Platelet Cloaking” and Neutrophil Extracellular Traps

**Figure 3:**
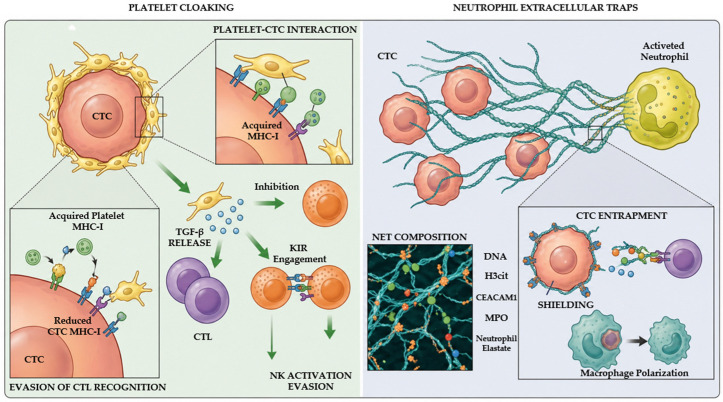
Extrinsic protection mechanisms: platelet cloaking and neutrophil extracellular traps. The left panel illustrates platelet-mediated cloaking as a key extrinsic immune evasion strategy adopted by CTC. Platelet adhesion to the CTC surface provides a physical shield that masks tumor-associated antigens, facilitates the transfer of platelet-derived MHC-I molecules, and promotes the release of immunomodulatory factors such as transforming growth factor-β (TGF-β). Collectively, these mechanisms impair cytotoxic T lymphocyte (CTL) recognition and inhibit natural killer (NK) cell activation, thereby enhancing immune escape and CTC survival. The right panel depicts the formation of neutrophil extracellular traps (NET), consisting of extracellular DNA fibers decorated with histones and granule-associated proteins, which entrap CTC within the circulation. NET-mediated sequestration provides a protective physical barrier against immune effector cells, facilitates interactions with immune and stromal components, and may promote macrophage polarization toward a pro-tumorigenic phenotype, ultimately favoring metastatic dissemination.

In addition to intrinsic mechanisms, CTCs exploit “extrinsic” protection strategies, interacting with blood components to create a physical and functional barrier against the immune system (Figure 3). One of the most studied mechanisms is “platelet cloaking,” the coating of CTCs by platelets [56]. The study by Placke et al. demonstrated in vitro that tumor cells are rapidly coated by platelets forming heterotypic aggregates with a “pseudonormal” phenotype due to the transfer of platelet MHC class I onto the tumor cell surface, preventing missing self-recognition by NK cells. This transfer, in fact, suppresses NK cytotoxicity and IFN-γ production [56]. More recently, it has been confirmed that activated platelets aggregate and encapsulate CTCs forming tumor microthrombi containing fibrin clots that act as protective barriers, interacting with NK cells, macrophages, neutrophils, and T cells to facilitate metastasis [57]. A 2011 study demonstrated both in vitro and in vivo that platelet TGF-β and direct platelet–CTC contact synergistically activate TGFβ/Smad and NF-κB pathways in tumor cells, inducing a transition to an invasive mesenchymal phenotype. Indeed, ablation of TGFβ1 expression induced exclusively in platelets protected mice from pulmonary metastasis formation in vivo [58]. Cluxton et al. described two other mechanisms by which platelets help tumor spread: (1) an immune decoy mechanism whereby platelets induce the release of soluble NKG2D ligands from tumor cells suppressing NK degranulation and IFN-γ production; (2) a TGF-β-mediated mechanism of suppressing the CD226/CD96-CD112/CD155 axis as an NK anti-tumor pathway [59]. Sun et al. identified by scRNA-seq and multiplex immunofluorescence that direct platelet adhesion to CTCs upregulates the inhibitory checkpoint CD155 suppressing NK cytotoxicity exclusively through the CD155-TIGIT interaction; blocking TIGIT in vivo restored NK immunosurveillance and markedly reduced metastasis [60]. Specifically for CRC, Plantureux et al. demonstrated that platelets extravasate into the tumor microenvironment and interact with tumor cells in a cadherin-6-dependent manner, generating chimeric microparticles (iMP) that in the circulation induce EMT and promote metastasis, while locally they recruit macrophages with anti-tumor activity [61]. Furthermore, in 2023, colon CTC lines were used to study CTC–platelet crosstalk, demonstrating that conditioned medium from CTCs induces platelet aggregation and activation, while co-culture with platelets increases the expression of genes involved in invasiveness (MMP2, MMP9, VEGFA) and maintains mesenchymal markers in CTCs [62]. In vivo, it has also been demonstrated that podoplanin expressed on tumor cells in mice induces the release of TGF-β from platelets, thus promoting EMT and metastasis formation [63].

Concurrently, Neutrophil Extracellular Traps (NETs) represent an additional mechanism supporting CTC survival. NET are filamentous structures composed of DNA and antimicrobial proteins released by activated neutrophils [64]. In the tumor context, NETs can trap CTCs in the circulation or in capillaries, facilitating endothelial adhesion and metastasis formation; protect CTCs from attack by immune cells; and promote a pro-inflammatory and pro-thrombotic state favorable to metastatic colonization [64]. In 2017, Najmeh et al. demonstrated, through an intra-abdominal sepsis mouse model (mimicking the post-operative inflammatory environment), that NETs sequester CTCs through β1-integrin-mediated interactions both in vitro and in vivo [65]. Yang et al. demonstrated that NET-DNA acts as a chemotactic factor attracting tumor cells. Briefly, the transmembrane receptor CCDC25 on tumor cells recognizes NET-DNA and activates the ILK-β-parvin pathway, increasing cell motility and thus metastatic capacity [66]. Furthermore, it has been shown that NETs wrap and coat tumor cells, shielding them from CD8+ T lymphocyte and NK cytotoxicity by physically obstructing contact between immune cells and tumor targets [67].

In CRC, high levels of NETs have been associated with unfavorable prognosis and increased risk of metastasis, suggesting a key role for these structures in disease progression. In 2020, Rayes et al. identified NET-associated CEACAM1 as an essential element for NET-CTC interaction in CRC [68]. Conversely, Stehr et al. detected citrullinated NETs (H3cit+) in 44% of colon cancer tissues (37/85 patients), with a significant association with high histological grades and lymph node metastases. In vitro, purified NETs induced filopodia formation, cell motility, and EMT in CRC cell lines, promoting the upregulation of vimentin, fibronectin, ZEB1, and Slug and inducing the downregulation of E-cadherin and EpCAM [69]. Lactate dehydrogenase A (LDHA) expression also appears to play a role in NET-promoted metastasis. Li et al. demonstrated, in fact, that inhibition of LDHA or NETs formation effectively inhibited NET-induced liver metastasis in vivo, suggesting an active role of LDHA regulation through the PI3K/AKT pathway in inducing EMT in CRC cells [70]. In 2026, Pan et al. demonstrated how *Escherichia coli* recruits RIPK2 in neutrophils, promoting NET formation via p-MLKL, which in turn stabilize STAT3-dependent enhancer–promoter loops in CRC cells, reinforcing Tns1 transcription and facilitating liver metastasis [71]. Regarding the prognostic value of NET in CRC, a systematic meta-analysis on 5202 cancer patients confirmed that high NET levels are significantly associated with worse OS (HR 1.80; 95% CI 1.35–2.41) and DFS (HR 2.26; 95% CI 1.82–2.82), independent of the sample source (tissue or blood) [72]. However, in a study of 1927 CRC patients across three independent cohorts, it emerged that intra-tumoral Cit-H3+ NET densities were not associated with survival, whereas a higher density of CD66b+ granulocytes was associated with longer CRC-specific survival (multivariate HR 0.53; 95% CI 0.38–0.73), suggesting that the prognostic role of NETs in CRC may be more complex than initially hypothesized [73].

To provide clearer synthesis, CTCs exploit a coordinated network of extrinsic defense mechanisms, such as platelet cloaking and NET entrapment, that synergistically shield them from immune destruction during hematogenous transit: platelets confer a “pseudonormal” phenotype through MHC-I transfer, suppress NK cell recognition via soluble NKG2D ligand release and TIGIT-CD155 engagement, and induce EMT through TGF-β signaling, while NETs physically sequester CTCs from cytotoxic lymphocytes, promote endothelial adhesion via β1-integrin and CCDC25-mediated chemotaxis, and drive EMT and metastatic colonization, particularly in CRC, where NET-associated CEACAM1 and platelet-derived chimeric microparticles further orchestrate the metastatic cascade toward the liver, although the prognostic significance of intra-tumoral NETs remains debated, with evidence suggesting that granulocyte infiltration density may be a more reliable prognostic indicator than NET density alone.

## 3. CTC Cellular Interactions in the Circulation

It is now clear that CTCs do not travel in the bloodstream as isolated entities but interact dynamically with various components of the immune system and beyond [79]. These interactions represent a crucial step in the metastatic process, influencing both CTC survival and their ability to colonize distant sites [80]. In particular, the dialog between CTCs and immune cells can have a dual role: on one hand, it promotes the recognition and elimination of tumor cells; on the other hand, it can favor immune evasion mechanisms and support the formation of pro-metastatic niches [8,81]. Understanding the nature of these interactions is therefore essential to clarify the fate of CTCs in the circulation and their contribution to CRC progression.

### 3.1. The Liver Microenvironment in Colorectal Cancer Metastasis

The liver is the predominant metastatic site in CRC, with approximately 20% of patients presenting with distant metastases at diagnosis [82]. This is partly due to the anatomical colon–liver relationship via the portal system [83].

Portal circulation exposes CTCs to a tolerogenic environment rich in Kupffer cells and sinusoidal endothelial cells [84].

A growing body of evidence, particularly from CRC models, highlights the role of the liver microenvironment in shaping CTC–immune interactions. Zeng et al. showed that the hepatic immune microenvironment directly influences colorectal metastasis, with resident liver cells (Kupffer cells, HSCs, LSECs) being co-opted to create an immunosuppressive environment favorable to tumor colonization [85]. Mourad et al. reported that the liver develops a highly tolerogenic and immunosuppressive niche, characterized by myeloid cell accumulation, T-cell depletion, and systemic immune suppression [86]. Using single-cell RNA-seq and spatial transcriptomics, Wu et al. demonstrated substantial spatial remodeling of the hepatic metastatic microenvironment, with reprogramming of M2-like macrophages [87]. Together, these mechanisms may facilitate immune evasion and the establishment of pre-metastatic niches. Li et al. showed that FGL1, secreted by tumor cells and hepatocytes, facilitates CRC progression in the liver by reducing T cell infiltration, with tumor-associated macrophages (TAMs) activating NF-κB in the liver microenvironment [88]. Zeng et al. confirmed that resident liver cells are co-opted by recruited cells (MDSCs, TAMs) to establish an immunosuppressive microenvironment suitable for tumor colonization [85].

Furthermore, neutrophil recruitment and the formation of NETs are also key processes in the CRC liver metastasis cascade. Yang et al. demonstrated that NETs trap CRC cells in the liver, stimulate tumor proliferation and invasion through an IL-8-mediated positive loop, and that NET digestion with DNase I reduces liver metastases [89]. Moreover, the transmembrane receptor CCDC25 on tumor cells acts as a receptor for NET-DNA, activating the ILK-β-parvin pathway to increase cell motility, thereby enabling NETs in the liver to act as a chemotactic factor for tumor cells [66]. Xu et al. identified distinct neutrophil subpopulations, showing that pro-tumor neutrophils, stimulated by tumor-derived GM-CSF and CXCR2 ligands, release NETs that promote tumor growth, suppress immune responses, and prime the liver for metastasis [90]. Zhou et al. also showed that FGL2 in TAM-derived extracellular vesicles promotes NET formation and tumor stemness in the CRC liver metastatic niche [91]. Meanwhile, Jiang et al. identified a Prok2+ neutrophil subpopulation infiltrating the pre-metastatic and metastatic liver, expressing PD-L1 and suppressing macrophage phagocytosis while promoting T-cell exhaustion [92]. In this context, it is important to emphasize the dual role of Kupffer cells in the hepatic metastatic cascade. On one hand, Kupffer cells represent the first line of defense against CTCs, being able to phagocytose and destroy them through innate immunity receptors such as Dectin-2 [93]. On the other hand, once reprogrammed by tumor microenvironment signals, Kupffer cells transform into pro-metastatic effectors, contributing to the creation of the immunosuppressive niche through the secretion of VEGF, PGE2, IL-6, and IL-10, and the expression of immune checkpoints such as VSIG4-CD5, which limits T-cell activation against poorly immunogenic metastatic clones [94]. This functional transition of Kupffer cells, from anti-tumor sentinels to facilitators of metastatic colonization, reflects the broader plasticity of the hepatic immune microenvironment and represents a potential therapeutic target to disrupt the metastatic cascade in CRC [95].

In summary, the liver microenvironment undergoes a progressive shift from immune surveillance to metastatic permissiveness in CRC: Kupffer cells transition from anti-tumor sentinels, capable of CTC phagocytosis via Dectin-2, to pro-metastatic effectors secreting immunosuppressive cytokines and expressing inhibitory checkpoints such as VSIG4-CD5, while NETs and recruited immunosuppressive cells (MDSCs, TAMs) cooperatively remodel the hepatic niche into a tolerogenic sanctuary that facilitates CTC colonization, underscoring the therapeutic potential of targeting hepatic immune cell plasticity to disrupt this cascade.

### 3.2. CTC-PBMC Clusters: Cellular Crosstalk and Pro-Metastatic Niches

An emerging aspect in CTC biology is their ability to form clusters [96]. It has been widely demonstrated that CTC clusters have a metastatic potential 23–100 times higher than single CTCs [97]. In a clinical study conducted on 103 CRC patients, Divella et al. noted that the presence of clustered CTCs was significantly associated with elevated levels of TGF-β and CXCL1 and reduced overall survival. Taken together, these results showed that CTC clustering represents a negative prognostic factor [98].

**Figure 4:**
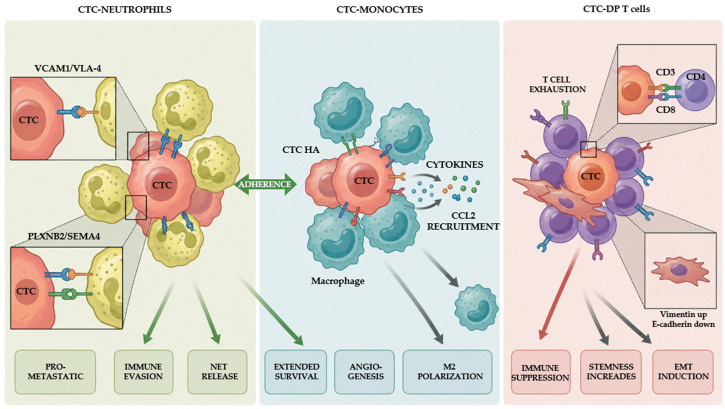
Heterotypic CTC clusters and their pro-metastatic role. The schematic illustrates the main heterotypic CTC cluster configurations involved in metastatic progression, including interactions with neutrophils, monocytes/macrophages, and double-positive T cells. These multicellular aggregates promote tumor cell survival in circulation, immune evasion, epithelial–mesenchymal transition (EMT), stemness acquisition, and metastatic seeding through cell–cell adhesion mechanisms, cytokine signaling, and immunosuppressive pathways. CTCs: circulating tumor cells; DP: double-positive; HA: hyaluronic acid.

CTC clusters can be divided into homotypic, composed solely of tumor cells, and heterotypic, arising from the aggregation of tumor cells with peripheral blood mononuclear cells (PBMC), including lymphocytes and monocytes [99] (Figure 4). These cellular aggregates are not simple passive associations but highly organized structures in which intense molecular crosstalk is established [100]. For example, within CTC-PBMC clusters, tumor cells can benefit from paracrine signals and direct cell–cell contacts that increase their survival in the circulation, protecting them from hemodynamic stress and immune attacks [101]. The study by Bobkov et al. demonstrated that hyaluronic acid (HA) mediates CTC clustering independently of adherens junctions and that HA acts as an anchoring platform to promote heterotypic cluster formation by recruiting immune cells and increasing CTC survival under hemodynamic stress [102].

Moreover, PBMC associated with CTCs can actively contribute to creating a favorable microenvironment for metastatic dissemination [99]. A study characterizing CTC-leukocyte clusters analyzed blood samples from 70 breast cancer patients and found that CTC-neutrophil clusters were the most frequent population with greater metastatic competence compared to single CTC [103]. Furthermore, an in vitro study highlighted that neutrophils form microtentacles (McTNs) composed of detyrosinated/acetylated α-tubulin and vimentin, which facilitate heterotypic cluster formation [104]. According to Ju et al., CTC-neutrophil clusters help CTCs survive in the hostile vascular environment by enhancing their metastatic capabilities [104]. Conversely, Spiegel et al. demonstrated in murine models that CD11b+/Ly6G+ neutrophils promote metastasis by inhibiting NK cell function and facilitating extravasation through the secretion of IL-1β and metalloproteinases [105].

Scholten et al. not only noted that over 75% of CTC-positive blood samples from advanced breast cancer patients contained heterotypic CTC–white blood cell (WBC) clusters but also that a rare subpopulation of CD4+CD8+ double-positive T (DPT) cells was enriched 140-fold in CTC clusters compared to their frequency in WBC. Moreover, the interaction between CTCs and DPT cells, mediated by the VCAM1/VLA-4 axis, conferred unique exhaustion and immunosuppression characteristics on tumor cells [106].

Monocytes and certain lymphocyte subpopulations can release cytokines, chemokines, and growth factors that promote the phenotypic plasticity of CTCs, including EMT, thus increasing their invasiveness and extravasation capacity. Schuster et al., through computational ranking, identified the transmembrane protein Plexin-B2 (PLXNB2) as a key mediator of CTC-monocyte clusters. PLXNB2 was found to be enriched in CTC clusters compared to single CTC from advanced breast cancer patients. In vivo, loss of PLXNB2 reduced the formation of both homotypic and heterotypic clusters, reducing spontaneous metastasis, whereas the interaction of PLXNB2 with SEMA4C (on tumor cells) and SEMA4A (on monocytes) promoted homotypic and heterotypic clustering, respectively, guiding lung metastasis [107]. Amin et al. demonstrated through an in vitro study that tumor cell–monocyte crosstalk induces the early release of TNF-α and IL-6, thus promoting migration, invasion, colony formation, and EMT [108]. Specifically for CRC, a study conducted by Wei et al., both in vitro and in vivo, highlighted that CD163+ TAMs induce EMT through the secretion of IL-6 and activation of the JAK2/STAT3/miR-506-3p/FoxQ1 axis, increasing the proportion of mesenchymal CTCs, which in turn produce CCL2 that recruits macrophages, creating a positive feedback loop [109].

A further relevant element is the ability of CTC-PBMC clusters to act as pre-conditioned metastatic “seeds.” These aggregates can facilitate adhesion to the endothelium and subsequent engraftment in secondary tissues, acting as functional units capable of rapidly establishing a pro-metastatic niche. In this context, the crosstalk between CTCs and immune cells not only favors survival in the circulation but also directly contributes to the efficiency of the metastatic process [110]. Vrynas et al. demonstrated through microfluidic models, which mimic human capillary bifurcations, how CTCs interact with capillary beds, and it emerged that CTCs release extracellular vesicles (EVs), termed shearosomes, capable of polarizing monocytes towards pro-tumoral M2 macrophages and altering endothelial barrier integrity, thus influencing the metastatic cascade [111].

Notably, while the biological principles of heterotypic CTC clustering are broadly applicable across solid tumors, the majority of direct experimental evidence on CTC-immune cell clusters derives from breast cancer models [102,103,106,107] and, to a lesser extent, from lung cancer models [108]. In CRC, direct evidence on heterotypic clustering remains more limited and further CRC-specific studies are warranted to validate whether the clustering mechanisms identified in breast and lung cancer are similarly operative in the colorectal setting and to define their prognostic and therapeutic relevance in this disease context.

In conclusion, CTC clusters (homotypic or heterotypic) represent highly organized metastatic units in which tumor cells exploit molecular crosstalk with blood-borne immune cells, particularly neutrophils, monocytes, and rare T cell subpopulations, to enhance survival under hemodynamic stress, evade NK cell-mediated killing, induce EMT through paracrine signaling loops (IL-6/JAK2/STAT3, TNF-α, TGF-β), and precondition distant endothelial sites for metastatic colonization via mechanisms such as shearosome release and integrin-mediated adhesion, establishing CTC clustering as both a key driver of metastatic efficiency and a negative prognostic factor in CRC (Table 2).

### 3.3. Macrophages and Myeloid-Derived Suppressor Cells

Among the immune populations involved in the interaction with CTCs, macrophages and myeloid-derived suppressor cells (MDSCs) play a central role in promoting tumor progression and immune evasion [118]. A study in prostate cancer showed that CTC are co-isolated with macrophage-like cells with TAM markers. Single-cell proteomic analysis and phenotyping revealed that macrophages promote epithelial–mesenchymal plasticity in tumor cells, conferring a “mechanical fitness” phenotype, characterized by softness and high adhesiveness, resulting in increased resistance to hemodynamic stress and facilitation of protective clustering [112].

Macrophages, particularly TAM-like, can interact with CTCs both directly and indirectly [119]. In circulation, monocytes can differentiate into cells with pro-tumoral characteristics, supporting CTCs through the secretion of soluble factors that promote survival, migration, and invasiveness [120]. Molecules such as IL-10, TGF-β, and angiogenic factors contribute to creating an immunosuppressive and pro-metastatic environment. As early as 1995, it was demonstrated that TGF-β potentiates the ability of macrophages to produce IL-10 in both healthy and tumor-bearing mice [121]. Subsequently, Li et al., using a 3D microfluidic assay, highlighted that TNF-α and TGF-β1 secreted by macrophages synergistically increase the speed and persistence of tumor cell migration through the extracellular matrix, in an MMP-dependent manner [113]. A recent study in CRC demonstrated that TGF-β builds a double immune barrier that blocks the recruitment of memory CD8+ T cells and instructs TAMs to express SPP1, promoting collagen decomposition and consequently the accumulation of TAMs and fibroblasts, driving immunotherapy resistance in liver metastases [114]. Harney et al. demonstrated using two-photon intravital microscopy that TIE2hi macrophages in the tumor microenvironment of metastases cause local and transient vascular permeability via VEGFA, thus allowing for tumor cell intravasation [115].

Furthermore, macrophages can facilitate CTC extravasation by modulating endothelial integrity and promoting tissue remodeling processes. The role of macrophages in tumor cell extravasation and pre-metastatic niche formation was clarified by Genna et al. through in vitro and in vivo studies, demonstrating, firstly, the formation of thin membranous connections between macrophages and tumor cells across the endothelial barrier and, secondly, that direct contact between macrophage and tumor cell is necessary to promote extravasation [116]. Häuselmann et al. also investigated the same aspect, demonstrating that monocytes recruited via endothelial E-selectin, induced by tumor CCL2, modulate endothelial VE–cadherin junctions, causing the opening of tight junctions and thus facilitating transendothelial migration of tumor cells [117].

MDSCs represent another key component in the negative regulation of the anti-tumor immune response [122]. These cells are known for their potent immunosuppressive activity, exerted through various mechanisms, including the production of arginase-1, nitric oxide (NO), and reactive oxygen species (ROS), which inhibit the function of T cells and NK cells [123]. The interaction between CTCs and MDSCs can therefore significantly reduce immune pressure, allowing tumor cells to survive and disseminate [124].

Moreover, MDSCs can directly contribute to the formation of pre-metastatic niches, favoring the recruitment of other immunosuppressive cells and the deposition of extracellular matrix components. This process creates a permissive environment for CTC engraftment in distant sites [125]. MDSCs recruited to pre-metastatic lungs, for example, produce IL-1β, which increases endothelial E-selectin expression and promotes tumor cell arrest [126]. Recent evidence also suggests that CTCs can actively modulate the recruitment and expansion of MDSCs, establishing a positive feedback loop that amplifies systemic immunosuppression [127]. A study of 38 HER2-negative metastatic breast cancer patients noted a higher frequency of Tregs and CD14+CD15- MDSCs in patients with detectable CTCs, suggesting that CTCs may be under the control of the immune system and that different immune evasion mechanisms are involved during their biological evolution [128].

In conclusion, macrophages and MDSCs act as cooperative architects of CTC immune evasion and metastatic dissemination: macrophages enhance CTC survival through direct contact-mediated mechanical reprogramming, paracrine secretion of TNF-α, TGF-β, and VEGFA that promote EMT, migration, and vascular permeability, and facilitation of extravasation via endothelial junction remodeling; while MDSCs suppress T cell and NK cell cytotoxicity through arginase-1, NO, and ROS production and prime pre-metastatic niches via IL-1β-driven endothelial activation. Together, these cells establish a self-reinforcing immunosuppressive circuit that shields CTCs throughout the metastatic cascade and represents a compelling target for therapeutic intervention in CRC.

Overall, macrophages and MDSCs not only protect CTCs from immune surveillance but actively enhance their metastatic potential, thus representing promising therapeutic targets in metastatic CRC.

## 4. The Role of the Immune System in CTC Elimination: Still Effective?

The immune system represents the first line of defense against CTCs, but its real effectiveness in controlling metastatic dissemination remains a matter of debate [129,130]. Preclinical evidence indicates that the majority of CTCs are eliminated in the bloodstream; however, clinical data demonstrate that a proportion of CTCs nonetheless manage to persist and give rise to metastases, suggesting that the immune system exerts a powerful but incomplete selective pressure [129,130].

Among the components of innate immunity, NK cells play a crucial role in the elimination of CTCs due to their ability to recognize cells with reduced MHC class I expression, a feature frequently observed in CRC cells [131]. NK induce direct cytotoxicity through the release of perforin and granzymes, in addition to secreting cytokines such as IFN-γ that amplify the immune response [131].

In vivo studies have provided direct evidence of the protective role of NK in CRC. In a murine xenograft model with HT29 colon carcinoma cells, mice lacking functional perforin in NK (pfp/rag2) developed lung metastases in 81% of cases (13/16) compared to 25% (5/20) of mice with functional NK (rag2), with an average number of metastases almost 4 times higher (789 vs. 210); computational modeling estimated that perforin-dependent NK cytotoxicity eliminates about 80% of CTC and forces residual tumor cells into a dormant state for at least 30 days [132]. Similarly, NK depletion with anti-asialo GM1 antibody in C57BL/6 mice increased lung metastases 10-fold after intravenous tumor cell inoculation, and prolonged NK suppression dramatically accelerated the development of spontaneous lung metastases without affecting primary tumor growth [133]. In murine models of CRC liver metastasis (MC38), NK depletion significantly increased metastatic burden, and CXCR3+CD49a+ NK proved to be the subpopulation with the highest cytotoxic capacity; conditional deletion of Cxcr3 in NKp46+ cells compromised NK accumulation and function in metastases [134]. Furthermore, STING signaling in macrophages promotes NK anti-tumor function in CRC liver metastases through 4-1BBL/4-1BB co-stimulation; NK depletion in wild-type mice increased metastatic burden, while no significant effect was observed in myeloid STING-deficient mice [135]. In vitro studies on patient-derived CRC organoids (PDOs) confirmed that most CMS2/CMS3 subtype PDOs are susceptible to NK-mediated lysis, and that MHC-I deficiency and NKG2D ligand expression on organoids facilitate cytotoxicity; pharmacological targeting of HIF1A/EPAS1 or TGF-βR1, or the use of anti-CEACAM1 antibodies, further enhanced PDO killing [136]. Allogeneic NK cells derived from cord blood demonstrated effective anti-tumor activity in primary and metastatic CRC suspensions, inducing tumor cell lysis, the conversion of monocytes into activated dendritic cells, and the activation of CD8+ and CD4+ T cells with a reduction in activated Tregs; a combination with R848 induced a pro-inflammatory shift with increased IFN-γ, IL-2, and IL-12p70 [137].

In summary, NK cells constitute the most effective innate immune barrier against CTCs in CRC, eliminating up to 80% of disseminated tumor cells through perforin/granzyme-dependent cytotoxicity and IFN-γ secretion, with specialized CXCR3^+^CD49a^+^ subpopulations and STING-dependent macrophage co-stimulation via 4-1BBL/4-1BB proving critical for hepatic metastasis control; however, tumor immune evasion strategies, including MHC-I modulation, NKG2D ligand shedding, and TGF-β signaling, allow a fraction of CTCs to escape this selective pressure, underscoring the therapeutic potential of NK cell-based approaches such as allogeneic cord blood-derived NK cells, pharmacological HIF1A/TGF-βR1 inhibition, and anti-CEACAM1 blockade to restore and enhance NK-mediated CTC clearance in CRC (Table 3).

## 5. Impact of CTCs on Systemic Immunity in CRC

**Figure 5:**
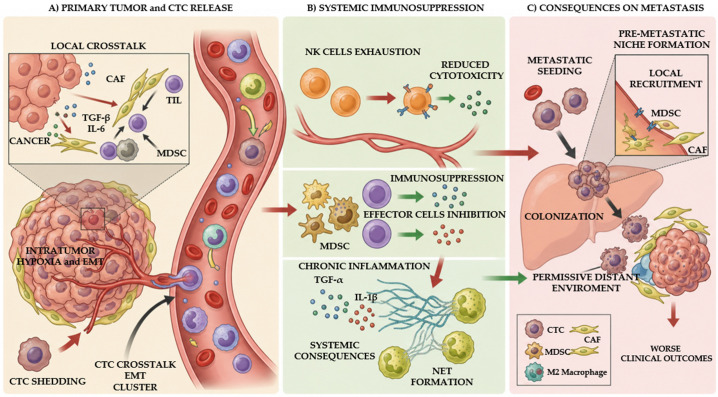
The impact of CTCs on systemic immunity: from local crosstalk to peripheral immunosuppression. A schematic representation that summarizes the dynamic interplay between primary tumor-derived circulating tumor cells (CTCs) and the immune system, highlighting the transition from local tumor microenvironment crosstalk to systemic peripheral immunosuppression. Tumor cell shedding and CTC release into the bloodstream promote immune remodeling through reduced natural killer (NK) cell activity, expansion of myeloid-derived suppressor cells (MDSC) and regulatory T cell (Treg), and chronic inflammatory signaling, including neutrophil extracellular trap (NET) formation. These systemic alterations establish a permissive pre-metastatic niche that supports metastatic seeding, colonization, and outgrowth at distant sites, ultimately contributing to increased metastatic burden and therapeutic resistance. CAF: cancer-associated fibroblast; EMT: epithelial–mesenchymal transition; TIL: tumor-infiltrating lymphocyte.

CTCs are not simply passive targets of the immune system but actively act as modulators of systemic immunity in CRC, inducing profound alterations in peripheral immune populations and creating an environment favorable to metastatic dissemination [23,81,138] (Figure 5).

In CRC patients, the presence of CTCs is associated with quantitative and functional remodeling of circulating immune cells. A study of 200 CRC patients (stages II–IV) demonstrated that CTC-positive patients (>3 CTCs) present significantly lower levels of NK cells (16.8% vs. 22.3%, *p* ≤ 0.05) compared to CTC-negative patients, with a redistribution of NK subpopulations characterized by an increase in immunosuppressive NK cells (8.8% vs. 5.0%, *p* ≤ 0.05) and immature CD16dimCD56bright cells (13.1% vs. 5.9%, *p* ≤ 0.05) at the expense of cytotoxic CD16+CD56dim cells (82.5% vs. 90.4%) [139]. These changes indicate a disorder in NK maturation and inhibition of their anti-tumor properties [139]. The impact of CTCs also extends to T lymphocytes. A study of 60 CRC patients revealed that in CTC-positive patients, the number of strong and moderate correlations between systemic immunological factors involving CD8+ is drastically reduced compared to CTC-negative patients (7 vs. 19), while correlations with Tregs are increased (5 vs. 3). In stages III and IV, a total disruption of correlations between systemic immune factors and proliferating tumor cells is observed, suggesting that the presence of CTCs, rather than the tumor stage itself, is associated with an imbalance of systemic and local immune factors [140]. Furthermore, a study on 299 CRC patients demonstrated a significant inverse relationship between the intensity of tumor lymphocytic infiltration and the presence of CTCs: CTCs were 1.4 times more frequent in tumors with weak lymphocytic infiltration compared to those with moderate or strong infiltration (76.5% vs. 56.3%, *p* = 0.019) [141].

CRC CTCs exert their immunomodulatory action through direct and indirect mechanisms. Gene expression analysis on manually isolated CTCs from CRC patients revealed a marked overexpression of CD47, an immune evasion mechanism that sends a “don’t eat me” signal to macrophages, together with a significant downregulation of several metabolic pathways, suggesting a dormant state of viable CTCs [44]. Recent studies have confirmed that 74.6% of CTCs in CRC express PD-L1, allowing them to escape elimination by T lymphocytes and persist in a dormant state in the bloodstream [38]. This PD-L1 expression is present across all stages, even in early phases and in patients with progression-free disease status, suggesting that PD-L1+ CTCs represent a dynamic biomarker of MRD [37].

CTCs also induce systemic inflammation through the production of TLR2 and TLR4 ligands. A preclinical study demonstrated that CTCs promote metastatic colonization of disseminated tumor cells by inducing systemic inflammation and neutrophil recruitment to pre-metastatic organs. Mechanistically, CTC-derived ligands for TLR2/4 induce the production of pro-inflammatory cytokines such as G-CSF and IL-6, which convert neutrophil function from tumor-suppressing to tumor-promoting. Moreover, CTCs induce the production of endogenous ligands for TLR2/4 such as S100A8, S100A9, and SAA3, which amplify the stimulatory effect on pro-inflammatory cytokine expression [142]. In metastatic CRC, the presence of CTC clusters is significantly associated with elevated circulating levels of TGF-β and CXCL1 and reduced overall survival. A study of 103 metastatic CRC patients demonstrated that circulating cytokine levels are differentially associated with the two CTC populations (single vs. clusters), with clusters representing a negative prognostic factor [98]. TGF-β, in particular, is one of the main immunosuppressive factors secreted by tumor cells, with elevated serum concentrations detected in CRC patients and associated with metastases [121]. A study on CRC also demonstrated that serum IL-17A levels correlate with the number of mesenteric CTCs and with disease-free survival. IL-17A promotes tumor cell motility, matrix digestion, and angiogenesis, while GM-CSF stimulates CTC elimination by enhancing host immunity; ablation of IL-17A and administration of rGM-CSF effectively suppressed the increase in CTCs and prevented metastasis in murine models [143].

Extracellular vesicles (EVs) derived from CRC tumor cells represent a key mechanism through which CTCs modulate systemic immunity. CRC EVs are enriched in TGF-β1 and induce phenotypic alteration of T lymphocytes into Treg-like cells through activation of the TGF-β/Smad pathway and inactivation of the SAPK pathway. Treg-like cells induced by CRC EVs have remarkable tumor growth-promoting activity in vitro and in vivo [144]. CRC EVs also modulate TAMs. A recent study demonstrated that TAMs initially recognize CRC exosomes as foreign entities, triggering a pro-inflammatory response; however, over time, the content of these phagocytosed exosomes reprograms TAMs into an anti-inflammatory and tumor-supportive phenotype, primarily through activation of the transcription factor NF-κB [145]. CRC EVs containing miR-372-5p can be phagocytosed by both CRC cells and macrophages, regulating PD-L1 expression through the PTEN/AKT/NF-κB pathway and inducing an immunosuppressive microenvironment that promotes CRC development [42]. CRC tumor EVs also suppress the CD28-CD80/86 co-stimulation pathway in tumor-infiltrating T lymphocytes and dendritic cells through miR-424 content, leading to resistance to immune checkpoint blockade. Tumor EVs modified with miR-424 knockdown enhanced the T-lymphocyte-mediated anti-tumor immune response in CRC models and increased the response to immune checkpoint blockade. Intravenous injections of modified tumor EVs induced antigen-specific immune responses and enhanced the efficacy of immune checkpoint blockade in CRC models mimicking advanced-stage aggressive disease [146]. Recent studies have also demonstrated that EVs derived from circulating tumor endothelial cells, enriched in mTOR, support G-CSF release and trigger phosphorylation of the downstream target of mTOR S6 (Ser235/236), ensuring tumor immunosuppression and metastatic growth through systemic and local expansion of immunosuppressive cells [147].

In vivo studies have confirmed that CTCs promote metastatic colonization by inducing systemic inflammation and neutrophil recruitment to pre-metastatic organs, and that neutrophil depletion effectively abrogates the pro-metastatic effect of CTCs [142].

A critical aspect of the impact of CTCs on systemic immunity concerns the disruption of physiological correlations between immune components. In CTC-positive patients, pathological correlations are observed, such as a moderate direct correlation between the number of activated T lymphocytes and Ki-67+ tumor cells, while the number of correlations between intra-tumoral lymphocytes and tumor cells expressing proliferation and EMT markers is drastically reduced compared to CTC-negative patients (4 vs. 10 in stage II, 1 vs. 9 in stage III, 2 vs. 9 in stage IV). This suggests that the presence of CTC destroys the functional architecture of the immune system, compromising both the local and systemic response [140].

In conclusion, CTCs in CRC function as active orchestrators of systemic immune reprogramming, simultaneously deploying multiple immunomodulatory strategies: direct immune evasion through CD47 and PD-L1 expression, induction of systemic inflammation via TLR2/4 ligands that convert neutrophils to a pro-tumoral phenotype, secretion of immunosuppressive cytokines (TGF-β, IL-17A) that reshape circulating NK and T-cell populations toward immature and regulatory phenotypes, and release of extracellular vesicles enriched in TGF-β1, miR-372-5p, and miR-424 that reprogram macrophages, generate Treg-like cells, and suppress co-stimulatory pathways, collectively dismantling the functional architecture of both local and systemic immunity and establishing a self-amplifying immunosuppressive circuit that primes pre-metastatic niches and facilitates metastatic colonization.

## 6. Therapeutic Implications and Future Directions

### 6.1. CTC–Immune System Interplay and Metastatic Dissemination

Current evidence clearly indicates that CTCs are not simply passive biomarkers of the disease, but dynamic players involved in a complex network of interactions with the immune system [8]. Firstly, CTCs play crucial roles in metastatic cascade and tumor immune evasion, and their molecular characterization has opened new avenues in understanding the biology of metastasis and the response to therapies directed against metastatic cells [8]. It is now also known that CTCs act both as biomarkers and active mediators of crosstalk with the immune microenvironment, influencing metastasis, immune evasion, and therapeutic resistance [138]. Furthermore, the controversial dialog between CTCs and the immune system has also been discussed in full detail, documenting both the anti-tumor activity and the immunosuppressive and pro-tumorigenic function mediated by NK cells, CD4 and CD8 T lymphocytes, Tregs, neutrophils, monocytes, macrophages, dendritic cells, and platelets [81].

In CRC, the fate of CTCs in the circulation is the result of an unstable balance between immunosurveillance mechanisms and immune evasion strategies, in which the dialog with innate and adaptive immune cells plays a determining role [80,129,140].

On one hand, effector components such as NK cells and CD8+ T lymphocytes contribute to the elimination of a significant proportion of CTCs, exerting a selective pressure that favors the survival of the most aggressive and immune-evasive clones [148]. For example, Ruggeri et al. demonstrated that CRC patients present early and pronounced alterations of NK cells in peripheral blood, with a reduced frequency of total CD56+ cells and impaired cytotoxic and cytokine responses. Furthermore, plasma from CRC patients induced similar dysfunctions in healthy donor NK cells, suppressing mTORC1 signaling and effector activity through a TNF-α deficiency-mediated mechanism [149]. On the other hand, CTCs are able to exploit and remodel the immune system to their advantage, through direct interactions with immune cells, the formation of heterotypic clusters, and the release of soluble factors and extracellular vesicles with immunomodulatory activity [79,99]. It has been demonstrated, in an in vitro and in vivo study, that small CRC-derived EVs promote tumor immune evasion by upregulating PD-L1 expression in TAMs through miR-21-5p and miR-200a, which synergistically regulate the PTEN/AKT and SOCS1/STAT1 pathways, resulting in decreased CD8+ T lymphocyte activity and increased tumor growth [150]. Regarding clusters, Wang et al. demonstrated in CRC that CTC-neutrophil clusters form at the vascular–immune interface of the primary tumor, guided by pericytes with high NNMT expression that activate the CXCL5/CXCR2 axis. Genetic and pharmacological inhibition of NNMT in pericytes eliminated CTC–neutrophil clusters and suppressed CRC liver metastases [151]. This dual role, already evident in the previous paragraphs, underscores how CTCs are positioned at the center of a bidirectional network of signals that promotes metastatic dissemination. Therefore, while PD-L1+ CTCs represent a promising biomarker, their predictive value in CRC requires further validation in large, prospective clinical studies. It is important to avoid implying immediate clinical readiness, as pre-analytical and analytical variables currently limit their routine use.

### 6.2. Therapeutic Implications: Neoantigens, Vaccines, and Emerging Technologies

**Figure 6:**
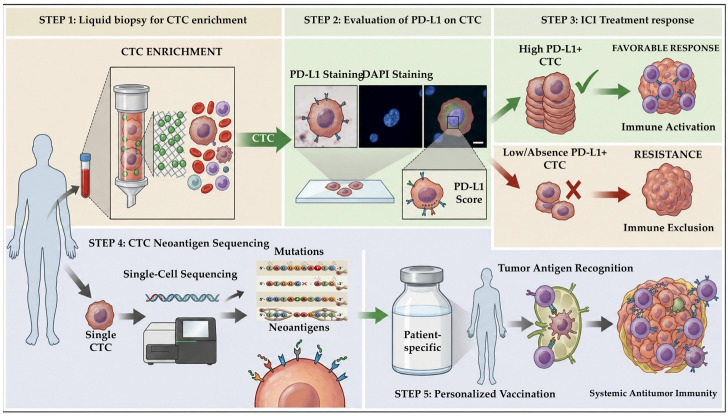
CTCs as predictive biomarkers for immunotherapy and platforms for personalized vaccination. A schematic overview of circulating tumor cells (CTCs) as predictive biomarkers for immunotherapy response and as a platform for personalized cancer vaccination. In the first track, liquid biopsy enables CTC enrichment, followed by PD-L1 characterization at the single-cell level to stratify patients undergoing immune checkpoint inhibitor (ICI) therapy. A high proportion of PD-L1-positive CTCs are associated with favorable response and tumor regression, whereas low or absent PD-L1 expression correlates with resistance and disease progression. In the second track, isolated CTC are subjected to single-cell genomic profiling (WGS/WES) to identify somatic mutations and derive patient-specific neoantigens. These neoantigens are then exploited for the development of personalized cancer vaccines, ultimately promoting T-cell activation, tumor antigen recognition, and durable systemic anti-tumor immunity.

The therapeutic implications of these processes are relevant, and the integration of CTCs into clinical pathways could improve patient stratification and therapy selection, particularly in the immunotherapeutic field [26,152]. The characterization of CTCs in terms of expression of immunoregulatory molecules, such as PD-L1, or mutational and neoantigen profiles, paves the way for personalized medicine strategies [153] (Figure 6).

In particular, the analysis of the mutational profile of CTCs has opened new perspectives for the identification of tumor neoantigens that could be exploited for therapeutic purposes [154]. High-resolution genomic techniques applied to single CTCs have demonstrated that these cells reflect the genomic complexity of the tumor, including driver mutations (e.g., APC, KRAS, PIK3CA) and structural variants, and can reveal clonal evolution in real time [155,156]. Neoantigens derived from tumor-specific somatic mutations represent ideal targets for immunotherapy due to their high immunogenicity and limited expression in healthy tissues. In CRC, recurrent “public” neoantigens such as KRAS G12D, KRAS G12V, and PIK3CA E545K have been identified, and their immunogenicity correlates with tumor mutational burden and distant metastasis [157,158]. Large-scale analyses have further defined the neoantigen landscape, distinguishing microsatellite-stable from MSI-high tumors [159]. Because CTCs are enriched for metastatic subclones, their neoantigen profiling offers a dynamic, non-invasive source for developing personalized therapeutic vaccines or adoptive T-cell receptor therapies [160,161,162,163]. Proof-of-concept clinical studies have shown that personalized neoantigen-reactive T-cell receptor-transduced T cells can induce objective responses in metastatic CRC patients [164], and that personalized mRNA vaccines co-administering MHC-I and MHC-II restricted neoantigens are safe and can generate specific T-cell responses, with synergistic effects when combined with immune checkpoint inhibitors (ICIs) [165,166,167]. Despite these advances, several challenges remain, including the difficulty of obtaining sufficient genetic material from rare CTCs, clonal heterogeneity, and the need to validate the true immunogenicity of identified neoantigens [158,168]. In this context, neoantigen profiling from CTCs, including the analysis of circulating tumor DNA (ctDNA) as a complementary analyte, could be used to develop personalized therapeutic vaccines or targeted adoptive immunotherapy strategies. ctDNA, which is often released by apoptotic or necrotic tumor cells including CTCs, provides a broader, averaged view of the tumor mutational landscape and can be analyzed with higher sensitivity for known driver mutations [159].

Integrating CTC analyses with multi-omics approaches and artificial intelligence (AI) could accelerate the development of personalized vaccination strategies [169,170].

In this direction, technological innovations are emerging. In 2024, Dong et al. developed the NICHE microfluidic platform (Nanoplatform for Interrogating Living Cell–Host–gene and Environment relationships) that integrates genetic and phenotypic profiling of single living CTCs, enabling on-chip co-culture of CTCs with immune cells and real-time quantification of phenotypic heterogeneities in response to ICIs. The generated predictive index, validated on 80 NSCLC patients, demonstrated an AUC of 0.906, significantly higher than current clinical benchmarks for predicting immunotherapy response [171].

### 6.3. Current Challenges and Future Directions

However, numerous challenges remain open. First of all, the discussion of EMT in the context of CTC biology must acknowledge that hybrid epithelial/mesenchymal (E/M) states, rather than a complete mesenchymal transition, may be especially critical for metastatic competence, CTC clustering, and immune evasion. EMT is now increasingly understood as epithelial–mesenchymal plasticity (EMP), a spectrum of intermediate phenotypes in which tumor cells retain epithelial features while simultaneously activating mesenchymal programs, enabling collective invasion, stem-like behavior, and immune escape [172,173].

As demonstrated by Mizukoshi et al., in CRC, tumor cell clusters expressing the hybrid E/M state seeded liver and lung metastases significantly more often than single tumor cells, and that inhibition of either E-cadherin or ZEB1 prevented metastatic colonization [174]. Huang et al. confirmed that unstable E/M-type CTCs had the strongest liver metastasis formation ability and that the proportion of E/M-type CTCs correlated with distant metastases in CRC patients [175]. These findings have direct implications for CTC detection technologies, as EpCAM-based methods such as CellSearch^®^ (Menarini Silicon Biosystems, Bologna, Italy) may fail to capture CTC subpopulations undergoing partial EMT with reduced EpCAM expression [176]. As highlighted by the international expert consensus, CellSearch^®^ is currently the only platform with high-level evidence for clinical use, although emerging technologies are promising [152]. In this context, artificial intelligence-driven image analysis platforms might further enhance the clinical utility of established technologies improving the accuracy, reproducibility, and standardization of CTC enumeration. In addition, AI-assisted approaches might facilitate the identification and characterization of rare circulating cell populations beyond conventional CTCs. [177]. Supporting this perspective, our group showed that automated CellSearch^®^ image analysis using ACCEPT software (University of Twente, v1.0.0-beta) improved the prognostic value of CTC enumeration in metastatic colorectal cancer and enabled the detection of circulating hybrid cells (CHCs), highlighting the potential of AI-assisted tools to uncover clinically relevant cellular phenotypes that may be overlooked by conventional approaches [178].

However, the ability to identify a broader spectrum of circulating tumor-derived cells does not fully overcome the limitations currently affecting the field. Indeed, key challenges still include improving detection sensitivity/specificity, harmonizing pre-analytical and analytical workflows, generating robust clinical evidence, and promoting the adoption of standardized methodologies across institutions [152]. The heterogeneity of CTCs, their rarity in peripheral blood, and the lack of standardized methodologies still limit their full clinical applicability. Saini et al. conducted a comparative study of seven CTC enrichment methods across five technologies in lung cancer, demonstrating that recovery rates varied significantly between methods (from 18% to 70%) and that EpCAM-based methods showed a dramatic reduction in recovery with low-EpCAM-expressing cells (from 70% to 1%), highlighting the need for phenotype-independent approaches [179]. Furthermore, the complexity of immune interactions, including the discrepancy between the peripheral compartment and the tumor microenvironment, requires integrated, multi-level approaches to be adequately understood [137].

In this context, future research directions should focus on the development of more sensitive and standardized technologies for CTC isolation and characterization, the integration of multi-omics data to more precisely define the immunological role of CTCs, the clinical validation of CTC-derived biomarkers in prospective studies, and the exploration of new therapeutic strategies aimed at interrupting the crosstalk between CTCs and the immune system. Ultimately, a better understanding of the interactions between CTCs and the immune system could not only clarify the mechanisms underlying metastasis but also open new opportunities for early and more effective therapeutic interventions in colorectal cancer (Table 4).

## 7. Conclusions

In conclusion, CTCs in CRC embody a precarious balance between immune surveillance and evasion, a dualism that determines their metastatic fate. Their survival in the circulation is based on a multifaceted strategy: on one hand, they finely modulate MHC-I expression to evade cytotoxic T lymphocytes without fully alerting NK cells, while on the other, they exploit immune checkpoints such as PD-L1, CD47, and FasL to silence effector responses. Added to these intrinsic mechanisms are extrinsic protections such as platelet cloaking, which transfers MHC-I and activates pro-invasive TGF-β signals, and NETs, which physically shield CTCs and favor their endothelial adhesion. In the bloodstream, CTCs do not travel in isolation but form heterotypic clusters with monocytes, neutrophils, and lymphocytes, fueling a crosstalk that increases resistance to mechanical stress and creates pro-metastatic niches, while macrophages and myeloid-derived suppressor cells amplify immunosuppression and facilitate extravasation. Concurrently, CTCs themselves remodel systemic immunity: they alter peripheral lymphocyte subpopulations, promote a chronic inflammatory state through TLR2/4 ligands, and release EVs enriched in TGF-β1, miR-372-5p, and miR-424 that convert immune cells into pro-tumoral phenotypes, destabilizing the physiological correlations between local and systemic immunity. In CRC, hepatic tropism via the portal circulation adds a further layer of complexity: CTCs encounter a tolerogenic liver microenvironment in which Kupffer cells exhibit a dual role, initially acting as phagocytic sentinels capable of eliminating CTCs through receptors such as Dectin-2, but subsequently undergoing reprogramming into pro-metastatic effectors that secrete VEGF, IL-6, and IL-10 and express immune checkpoints such as VSIG4, thereby facilitating the establishment of immunosuppressive pre-metastatic niches. Although NK cells and CD8+ T lymphocytes eliminate a substantial proportion of CTCs, selective pressure favors the emergence of increasingly resistant clones, often characterized by high PD-L1 expression. The dynamic analysis of PD-L1 on CTCs, together with neoantigen profiling of CTC-derived mutations and the development of functional platforms such as single-cell microfluidic technologies, is opening concrete perspectives for personalized immunotherapy, including neoantigen vaccines and adoptive T-cell receptor strategies. However, cellular heterogeneity, the discrepancy between peripheral and tumor microenvironment compartments, and the lack of standardization still limit its clinical application. Overcoming these technological challenges and validating CTC-derived biomarkers in large prospective studies will allow these cells to be transformed from simple passive indicators into active therapeutic targets, with the aim of interrupting the immunosuppressive dialog that sustains metastasis and improving treatment strategies in CRC.

## Figures and Tables

**Table 1:** Key mechanisms of immune evasion of circulating tumor cells in colorectal cancer (CRC).

Mechanism	Key Molecules	Effect on Immune Cell	Evidence in CRC
Downregulation of antigen presentation machinery	MHC-I, TAP1/2, β2-microglobulin, LMP7	Reduced recognition by CD8+ CTL; partial loss prevents “missing self” recognition by NK cells	[27,28,29,30,31,32,33,34]
Upregulation of immune checkpoints	PD-L1	Inhibition of T cell cytotoxicity via PD-1; T cell exhaustion	[35,36,37,38,39,40,41,42,43]
“Don’t eat me” signal	CD47	Inhibition of macrophage phagocytosis via SIRPα; cis-masking of SLAMF7	[44,45,46,47,48,49]
Fas counterattack	FasL	Induction of apoptosis in Fas-expressing T lymphocytes	[50,51,52]
Alternative Immune checkpoint	PD-L2	Suppression of T cell function; expansion of Tregs; expressed on tumor exosomes	[53,54,55]
Platelet cloaking	Platelet MHC-I, TGF-β, CD155	Physical shield; transfer of MHC-I to CTC; suppression of NK cytotoxicity via CD155-TIGIT; induction of EMT	[56,57,58,59,60,61,62,63]
Neutrophil extracellular traps (NETs)	NET-DNA, CEACAM1, histones	Physical barrier blocking CTL/NK contact; chemotaxis via CCDC25; induction of EMT	[62,63,64,65,66,67,68,69,70,71,72,73]

CEACAM1 (carcinoembryonic antigen-related cell adhesion molecule 1), CTL (cytotoxic T lymphocyte), EMT (epithelial-to-mesenchymal transition), FasL (Fas ligand), LMP7 (large multifunctional protease 7), MHC-I (major histocompatibility complex class I), NET (neutrophil extracellular trap), NK (natural killer), PD-1 (programmed cell death protein 1), PD-L1 (programmed death-ligand 1), SIRPα (signal regulatory protein alpha), SLAMF7 (signaling lymphocytic activation molecule family member 7), TAP1/2 (transporter associated with antigen processing 1/2), and Tregs (regulatory T cells).

**Table 2:** Heterotypic CTC clusters: composition and pro-metastatic mediators.

Cluster Type	Key Mediators/Crosstalk	Functional Effect	Model	Refs.
CTC–neutrophil	Microtentacles (detyrosinated tubulin, vimentin); IL-1β, MMPs	Enhanced survival, inhibition of NK cells, extravasation	Breast cancer	[103,104,105]
CTC–monocyte/TAM-like	Plexin-B2 (PLXNB2) with SEMA4A; TNF-α, IL-6, TGF-β, IL-10; JAK2/STAT3/miR-506-3p/FoxQ1	EMT, mechanical fitness, clustering, extravasation, M2 polarization	Breast cancer, prostate cancer, melanoma, CRC, lung cancer	[107,108,109,112,113,114,115,116,117]
CTC-DPT	VCAM1/VLA-4 axis	T-cell exhaustion, immunosuppression	Advanced breast cancer	[106]
CTC-PBMC	Hyaluronic acid (HA); shearosomes (extracellular [86,87,88] vesicles)	Clustering, protection from hemodynamic stress, M2 polarization, endothelial barrier alteration	Triple-negative breast cancer and lung cancer	[102,111]
CTC–macrophage	CCL2 loop, IL-6, JAK2/STAT3	Increase in mesenchymal CTCs, positive feedback recruitment	CRC	[109]

CCL2 (C-C motif chemokine ligand 2), CTC (circulating tumor cell), DPT (double-positive T cell), EMT (epithelial-to-mesenchymal transition), FoxQ1 (forkhead box Q1), HA (hyaluronic acid), IL (interleukin), JAK2 (Janus kinase 2), M2 (alternatively activated macrophage polarization state), McTN (microtentacle), MMP (matrix metalloproteinase), NK (natural killer), PBMC (peripheral blood mononuclear cell), PLXNB2 (plexin B2), SEMA4A (semaphorin 4A), STAT3 (signal transducer and activator of transcription 3), TAM (tumor-associated macrophage), TGF-β (transforming growth factor beta), TNF-α (tumor necrosis factor alpha), VCAM1 (vascular cell adhesion protein 1), and VLA-4 (very late antigen-4).

**Table 3:** Summary of preclinical and clinical evidence on NK cell activity against CRC CTC.

Model System	Condition	Effect	Key Findings	Refs.
Murine xenograft (HT29 CRC cell line)	pfp/rag2 mice (perforin-deficient NK) vs. rag2 (functional NK)	Lung metastases: 81% vs. 25%; mean number 789 vs. 210	Perforin-dependent NK cytotoxicity eliminates ~80% of CTC; forces dormancy for ≥30 days	[132]
C57BL/6 mice (intravenous CRC cells)	NK depletion with anti-asialo GM1	10-fold increase in lung metastases; accelerated spontaneous metastases	NK control both initial seeding and spontaneous dissemination	[133]
Murine CRC liver metastasis (MC38)	NK depletion; CXCR3 conditional deletion	Increased metastatic burden; CXCR3+CD49a+ NKs are key cytotoxic subset	CXCR3 required for NK accumulation and function in metastases	[134]
Murine CRC liver metastasis	STING signaling in macrophages; NK depletion	Increased metastatic burden in NK-depleted wild-type; no effect in myeloid STING-deficient	4-1BBL/4-1BB co-stimulation from macrophages promotes NK anti-tumor function	[135]
Patient-derived CRC organoids (PDOs)	CMS2/CMS3 PDO; HIF1A/EPAS1 or TGF-βR1 targeting; anti-CEACAM1	Enhanced NK-mediated lysis	MHC-I deficiency and NKG2D ligands facilitate killing; pharmacological targeting further potentiates lysis	[136]
Allogeneic cord blood NK + primary/metastatic CRC cells	NK alone or with R848	Tumor cell lysis; monocyte → DC conversion; CD8+/CD4+ T activation; Treg reduction	Combination with R848 increases IFN-γ, IL-2, IL-12p70	[137]

CEACAM1 (carcinoembryonic antigen-related cell adhesion molecule 1), CMS (consensus molecular subtype), CRC (colorectal cancer), CTC (circulating tumor cell), CXCR3 (C-X-C chemokine receptor type 3), DC (dendritic cell), EPAS1 (endothelial PAS domain protein 1), HIF1A (hypoxia-inducible factor 1-alpha), IFN-γ (interferon gamma), IL (interleukin), NK (natural killer), PDO (patient-derived organoid), pfp (perforin), rag2 (recombination activating gene 2), STING (stimulator of interferon genes), TGF-βR1 (transforming growth factor beta receptor 1), and Treg (regulatory T cell).

**Table 4:** Ongoing challenges and future directions for CTC-based immunotherapies in CRC.

Challenge	Clinical Impact	Potential Solution	References
Heterogeneity of CTCs	Underestimation of CTCs; missed aggressive subclones	Phenotype-independent isolation methods (e.g., size-based, microfluidic); multi-marker panels	[7,8,9,10,11,12,79,179]
Low frequency of CTCs in peripheral blood	Difficult to obtain sufficient material for molecular analysis	Pre-analytical enrichment; sensitive detection platforms (e.g., NICHE microfluidic)	[152,171]
Lack of standardization in isolation and analysis	Poor reproducibility across studies; no widely accepted cut-offs for PD-L1+ CTCs	International consensus protocols; automated systems; validation in large prospective cohorts	[152,180,181,182]
Discrepancy between peripheral CTCs and tumor microenvironment	CTCs may not fully reflect TME immune status	Integrated multi-omics approaches (scRNA-seq, proteomics) matched with tissue biopsies	[137,162,163]
Dynamic changes in PD-L1 expression on CTCs (under therapy pressure)	Risk of false negative/positive for immunotherapy guidance	Serial liquid biopsies; real-time monitoring; use of multiple antibody clones	[183,184,185,186]
Validation of neoantigen prediction from CTCs	Limited material for WES; low immunogenicity of common neoantigens	Single-cell sequencing; “public” neoantigen libraries (KRAS G12D, etc.); functional T cell assays	[157,158,159,160,161,168,187]
Clinical translation of CTC-based vaccination	Need for large trials; cost and logistics of personalized vaccines	mRNA vaccine platforms (personalized); neoantigen ranking by AI; combination with immune checkpoint inhibitors	[164,165,166,167,170]

AI (artificial intelligence), CRC (colorectal cancer), CTCs (circulating tumor cells), mRNA (messenger RNA), PD-L1 (programmed death-ligand 1), scRNA-seq (single-cell RNA sequencing), and WES (whole-exome sequencing).

## Data Availability

No new data were created or analyzed in this study. Data sharing is not applicable to this article.
